# No significant difference in salivary cortisol response on the Trier Social Stress Test-Online based on coffee consumption habits

**DOI:** 10.1186/s40359-024-01968-3

**Published:** 2024-09-13

**Authors:** Masaharu Ueno

**Affiliations:** Tobacco Academic Studies Center, 1-16-3, Yokokawa, Sumida-ku, Tokyo, 130-0003 Japan

**Keywords:** Coffee, Cortisol, Resilience, Trier Social Stress Test-Online

## Abstract

**Background:**

Coffee is widely consumed around the world. In Japan, it is a type of “Shikohin” (consumed for flavor, not nutrition). Several medical studies have reported the beneficial effects of coffee consumption, whereas others suggest that these beneficial effects on psychological aspects are marginal. The habit of consuming large amounts of caffeine through coffee may improve short-term resilience in stressful situations and may exhaust individuals in the long term. We hypothesized that people who habitually drink high amounts of coffee would have lower resilience scores and higher acute stress responses.

**Methods:**

Adult Japanese men completed a questionnaire that included a resilience scale and Shikohin consumption habits. Experimental participants were recruited from the survey respondents and classified into three groups based on their coffee consumption per day: No Coffee, Low Coffee, and High Coffee. All participants were asked to join the Trier Social Stress Test-Online (TSST-OL). Subjective stress and salivary cortisol concentrations was measured at eight time points during the experiment. There were 16 participants in each group for the analysis (mean age = 46.10 years, *SD* = 12.58).

**Results:**

Statistical analysis showed that both subjective stress and salivary cortisol concentrations significantly increased following TSST-OL exposure. However, there were no significant differences among the groups, and the hypotheses were not supported.

**Conclusions:**

This study demonstrated the effectiveness and stability of the TSST-OL. Additionally, coffee consumption habits were not significantly related to resilience scale scores or acute stress responses.

**Supplementary Information:**

The online version contains supplementary material available at 10.1186/s40359-024-01968-3.

## Background

The term Shikohin refers to luxury items and genußmittel, which has cultural nuances unique to the Japanese and includes alcohol, coffee, tea, and tobacco as major items [1]. Coffee is widely consumed as a type of Shikohin, and medical studies have shown that coffee has beneficial effects on several diseases, such as Parkinson’s disease, type 2 diabetes, and hepatocellular carcinoma [2, 3]. Coffee consumption has also been suggested to have a protective effect against the risk of depression [4–6]. In contrast, a previous study suggested that, for middle-aged and older women, the association between coffee intake and long-term psychological well-being and the maintenance of optimism was weak or minimal [7]. Another study reported no significant association between coffee intake and mental health among male workers in a Japanese car manufacturing company [8]. Those who drank two or more cups of coffee per day had a significantly higher proportion of mental unhealthiness scores than those who drank less than one cup per week.

Coffee contains caffeine and is one of the major sources of caffeine intake. It has been reported that among female college students, those experiencing anxiety and depression were associated with higher caffeine intake and that the source of most caffeine was coffee [9]. For caffeine intake in healthy adults, the European Food Safety Authority reported that a single intake of up to 200 mg and a habitual intake of up to 400 mg per day do not raise safety concerns [10]. This amount of caffeine can be calculated as 4–5 cups of coffee per cup (150 ml). Namely, coffee consumption in excess of this amount may be at risk mentally.

Regarding the relationship between caffeine, stress, and resilience, for example, if acute caffeine intake were to enhance the ability to concentrate, it would be a positive effect in stressful situations that require resilience, such as active coping. However, previous research has been inconsistent and dismisses such simplifications. One reason may be the importance of the situation in which coffee and caffeine are consumed. Acute coffee ingestion in the non-stressful situation increased salivary alpha-amylase concentrations and blood pressure, but not salivary cortisol concentrations [11]. Caffeine intake did not affect baseline cortisol, while it did further increase salivary cortisol concentrations in concert with mental stress exposure, especially in men [12]).

If caffeine enhances responses in concert with stress mechanisms, it may lead to increased resilience such as active stress coping in the short term and then to exhaustion in the long term. It has been previously indicated that resilience loads individuals [13, 14] and that negative aspects of resilience exist [15]. One previous study reported a significant negative correlation between resilience scale scores and the number of cups of coffee consumed per day [16]. Questionnaire measures of resilience tend to focus on overall cognitive and behavioral patterns, such as personality. The habit of repeated caffeine exposure through daily high coffee consumption may make individuals more active and exhausted in stressful situations. An animal study reporting that high dose of caffeine activated the HPA axis, while low and moderate doses did not modulate the hypothalamic-pituitary-adrenal (HPA) axis response to stress stimuli, partially supports this idea [17]. In laboratory experiments, high resilience is likely to be reflected by a lower stress response or faster recovery [18]. Thus, in this study, we expected that people who habitually drank high amounts of coffee would be less resilient and have a higher stress response.

This study aimed to determine whether participants classified by coffee consumption habits (No Coffee, Low Coffee, and High Coffee) showed group differences in resilience scale scores and cortisol concentrations after the Trier Social Stress Test-Online (TSST-OL). We hypothesized that the High Coffee group would have lower resilience scale scores than the Low Coffee and No Coffee groups. Additionally, we hypothesized that there would be no difference between the Low Coffee and No Coffee groups. In addition, we predicted that the salivary cortisol concentrations would increase after exposure to TSST-OL in all groups; that is, the High Coffee group would exhibit higher cortisol concentrations than the Low Coffee group, and the Low Coffee group would exhibit higher cortisol concentrations than the No Coffee group. A recent review indicated that dehydroepiandrosterone (DHEA) was associated with stress response and mental health because of its anti-glucocorticoid effects [19]. However, since no notable differences were detected in our previous experiments, we measured it for an exploratory analysis in this study [20, 21].

## Methods

### Online survey

The participants were recruited via an Internet research company (Macromill Inc., Tokyo, Japan). The online survey was conducted in two stages: screening and main survey. The completion of each survey was rewarded with cashable coupons, according to Macromill Inc.’s regulations. The online survey was conducted from November 16 to 29, 2023.

#### Screening survey

Demographic data, such as age and job type, were collected, as well as items related to the inclusion/exclusion criteria. The target population was “aged 20–69 years,” “male participants,” “did not suffer from any physical or mental illness at the time of the survey,” “people who owned a freezer,” “people who read the explanation of the online experiment and agreed to participate,” “people who agree to disclose research data from which their personal information has been excluded,” “could use a computer to participate in the experiment,” “lived in Tokyo, Saitama, Chiba, or Kanagawa prefectures,” “were able to come to Shinagawa station from their homes within 90 minutes,” “were able to talk online for more than three hours in a quiet environment using their home PC,” and “people who do not have bleeding from the mouth in daily life due to stomatitis or gingivitis.” Individuals who did not meet the criteria were excluded.

Exclusion criteria for Shikohin consumption habits, considering practical recruitment possibilities, were set as follows: (1) those who drank alcohol more than four times a week, (2) those who smoked 15 or more cigarettes per day, and (3) those who drank energy drinks more than three times a week. In addition, exclusion criteria were established for the following elements that could affect cortisol dynamics: (4) To exclude patterns such as high coffee intake due to high stress levels, we asked about daily stress and excluded those who selected “I feel very much”; (5) We excluded those who habitually take medication; (6) individuals with a history of organ transplantation were excluded; and (7) individuals with a history of psychiatric or physical illness were excluded. Examples of diseases include diabetes, atopic dermatitis, myocardial infarction, stroke, Alzheimer’s disease, epilepsy, Parkinson’s disease, depression, adjustment disorders, alcoholism, anthropophobia, and social anxiety. As a default setting in the survey company, research and advertising agency occupations were excluded from the survey’s target population.

#### Main survey

The main survey asked participants “how many cups of coffee they drink per day,” “how many days per week they drink coffee,” “how long they have been drinking coffee in their lives,” “how much sleep they get per day,” and “how often they usually exercise.” Based on approximately 150 mL per cup, the participants were asked how many cups of instant coffee, regular coffee, or canned coffee they drank per day. Coffee-related products, such as cafe lattes and cafe au lait, were not targeted in this study. Additionally, decaffeinated coffee was not included in this study to determine the contribution of caffeine.

Based on the survey results of coffee consumption habits (number of cups of coffee consumed per day), the participants were classified into three groups: High Coffee, Low Coffee, and No Coffee. The High Coffee group consisted of those who drank four or more cups of coffee per day, the Low Coffee group consisted of those who drank one to three cups of coffee per day, and the No Coffee group consisted of those who did not drink coffee. This classification criterion was based on the report on the safety of daily caffeine intake [10]. The participants and judges in the TSST-OL (speech and math tasks) were blinded to the group classification. The experimenter (the first author) was aware of this. To establish the classification criteria, we referred to previous studies on coffee consumption and disease risk [6, 22, 23]. The amount per cup was based on the standards for Japanese coffee products.

In addition, we used four resilience scales of resource cognition and utilization [24], which included cognition (20 items, ω = 0.918) and utilization (29 items, ω = 0.960) of intrapersonal resources as well as cognition (20 items, ω = 0.963) and utilization (30 items, ω = 0.969) of environmental resources. The scales were developed based on the concept that resilience was defined by intrapersonal resources, including personality, and environmental resources, such as social support [25]. A similar scale dealing with resources was that of Friborg et al. [26]. The scales then evaluate resilience in terms of cognition (whether one is able to recognize these resources) and utilization (whether or not one is able to make effective use of these resources). We used all factors in the four scales and modulated the three items because they did not match the profiles of the participants in this study. The original description “at schools” was changed to “at schools and workplaces.” Each item was assessed on a five-point Likert scale ranging from 1 (strongly disagree) to 5 (strongly agree).

While delivering data, the staff of Macromill Inc. excluded unreliable respondents based on their response tendencies, for example, respondents who selected “1” for all the questions. After the main survey, the staff at Macromill Inc. initiated recruitment calls for those who met the criteria for participation in the online experiment. To exclude people who consume other Shikohin (alcohol, tobacco, and energy drinks), recruitment was based on the priorities of Shikohin consumption habits created by the author, with adjustments for age (Supplementary Table 1). Individuals who clearly displayed problems with the way they talked or the content of their conversations during the recruiting call were excluded. The participants were assigned as equally as possible according to age within each experimental group.

### Online experiment (TSST-OL)

**Fig. 1:**
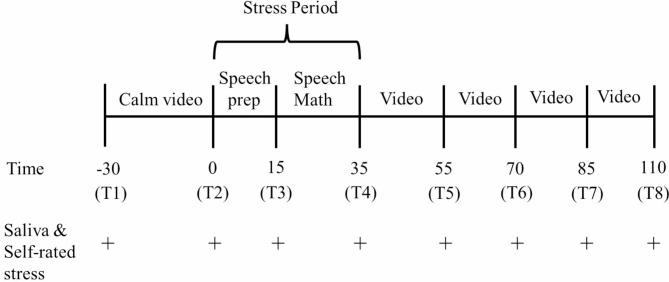
Timeline of the TSST–OL protocol. The time interval corresponds to the horizontal axis of the results graph (Figs. 2 and 3)

Prior to the TSST-OL, the experimenter and participant conducted a video call to check the communication conditions using Zoom™ (https://zoom.us/). Additionally, an explanation of the experiment was provided, which included restrictions (no eating, drinking, heavy exercise, smoking, and brushing teeth 1 h prior to the experiment; waking up before 9:00 am the day before and on the day of the experiment; no alcohol consumption, reporting of dental treatment and medication during those two days; and no caffeinated beverage consumption for 12 h prior to the experiment), and ethical considerations were explained. Participants were mailed saliva collection tools, a manual on saliva collection procedures, and the address of the sample collection site. On the day of the TSST-OL, the breakout and waiting room functions of Zoom™ were used to set up the experimenter and two judges in the main and breakout rooms, respectively. The experimenter was always in the main room, the two judges were always in the breakout room, and only the participant moved between rooms. The experiments were conducted between 14:15 and 21:00. Figure 1 shows an outline of the TSST-OL procedure. The time notation in Fig. 1 corresponds to the horizontal axis of the results graph (see below).

The TSST experiment was performed using a modified procedure described by our previous studies [20, 21], based on the work of Meier et al. [27]. During the TSST-OL session, the self-rating of stress and saliva collection was conducted at eight time points: (1) before the baseline period (T1), (2) pre-TSST (T2; 25 min after T1), (3) after speech preparation (T3), (4) after completion of the speech and math tasks (T4), (5) post-TSST (T5; 15 min after T4), (6) recovery period 1 (T6; 10 min after T5), (7) recovery period 2 (T7; 10 min after T6), (8) recovery period 3 (T8; 20 min after T7).

To measure the concentrations of cortisol and DHEA in saliva, participants were asked to collect approximately 1.0 ml of saliva in microtubes (2 ml), which were frozen (approximately − 20°C). We used Cryovial (2 ml, Salimetrics, LLC, USA) and Saliva Collection Aid (Salimetrics, LLC, USA) for saliva collection. For self-rating of stress, participants were asked to rate “how stressed did you feel.” Each rating was on a five-point scale, with 1 =“not at all stressed” and 5 = “highly stressed.” Self-rated stress was measured immediately after saliva collection.

During the TSST-OL procedure, after the participants entered the main room in Zoom™, the experimenter checked the video and audio, confirmed that they could withdraw their consent to participate at any time during the experiment, and that they had not eaten or drank during the previous hour. Next, the experimenter explained the saliva sample collection procedure, requested that participants collect their first saliva sample, and asked them to measure their subjective stress (T1). The participants were then asked to consume two pieces of glucose (5 g in total) to control their cortisol concentrations [27, 28]. Subsequently, the participants were asked to watch a video of nature for 25 min in a comfortable sitting position, after which they were asked to collect a second saliva sample (T2).

The experimenter told the participants that they were about to give a speech and that they had 5 min to prepare it. The experimenter explained the details of the task to the participants, which was to assume that they had been called for a job interview for a position of interest and to give a speech about themselves that would demonstrate convincingly that they would be suitable for the position. Participants were informed that they could make memos but not look at them during their speeches. The experimenter explained that the speech would be recorded, compared, and evaluated by others. After 5 min, the experimenter asked the participants to collect a third saliva sample (T3). The participants were then asked to stand up, step back, and adjust their positions so that the screen displayed them from the waist up. Participants were guided to a breakout room for the speech and mental arithmetic tasks.

In the breakout room, two judges wearing white lab coats confirmed that the participants had no audio or visual problems and asked them to perform their speeches (5 min). The judges were male–female pairs and were not informed of the groups to which the participants were assigned or the hypotheses. During the speech, the judges ensured that there was no expression or reaction, and if the participant remained silent for more than 20 s, they were instructed to continue. After 5 min, the participants received explanations about the mental arithmetic task. During the mental arithmetic task (5 min), the participants repeatedly told the judges their answers to a calculation task involving repeatedly subtracting 13 from 938. If the participants made a mistake in the calculation or remained silent for 30 s, they were asked to start from the beginning. Depending on the participants’ performance, the difficulty level of the task was adjusted to either a more difficult level (subtracting 17 from 938) or two easier levels (subtracting 7 or 3 from 938).

After the math task ended, the participants were led back to the main room, where the experimenter instructed them to perform saliva sampling (T4). The participants were then asked to view a video of nature for 15 min, after which saliva sampling was performed (T5). Saliva collection and subjective stress measurements were conducted 10, 20, and 40 min after T5. Between the time points, the participants were asked to watch a video of scenes from nature. After T8, participants were debriefed.

The online experiment was conducted from December 18, 2023, to February 17, 2024. All the participants brought frozen saliva samples to a collection site in Tokyo on February 18, 2024. Samples were stored in a freezer (-20 °C) for one day and then transported from the collection site to Yanaihara Institute Inc. (Shizuoka, Japan) at freezing temperature (-79.2–81.2 ℃) by a professional transporter for biological samples (SAROUTE Co., Ltd.). In Japan, intact saliva samples cannot be transported by postal services or ordinary delivery companies without virus inactivation treatments owing to the COVID-19 pandemic.

Hormonal assays of the saliva samples were conducted by Yanaihara Institute, Inc. (Shizuoka, Japan). The Cortisol (saliva) EIA Kit (Cat. No. YK241; assay range: 0.012–3.000 µg/dL, Yanaihara Institute Inc., Shizuoka, Japan) was used. The intra- and inter-assay coefficients of variation were 3.8% and 8.7%, respectively. The DHEA (saliva) EIA Kit (Cat. No. YK290; assay range: 22.222–5400 pg/mL, Yanaihara Institute Inc., Shizuoka, Japan) was also used. The intra- and inter-assay coefficients of variation were 6.2% and 4.4%, respectively. Hormone assays were performed according to the instructions provided with the kit after centrifugation of the saliva samples at 3000 rpm for 10 min. Saliva samples were measured in duplicate; if one measured value was below the detection limit (0.012 µg/dL or 22.222 pg/mL), the value was rejected, and the other value that could be detected was adopted. If both measurements were below the detection limit, the individual was excluded from analysis. Those who completed the experiment in approximately 2.5 h were given rewards equivalent to JPY 18,000 via Macromill, Inc.

### Participants and sample size rationale

We used G*Power 3.1.9.7 to estimate the required sample size for two-way repeated measures analysis of variance (ANOVA) and investigate the hypothesis in the TSST-OL. We set the effect size to be smaller (f = 0.175) based on previous studies on TSST-OL for adults [27]. We calculated the required sample size using “ANOVA: Repeated measures, within–between interaction” mode (Effect size f = 0.175, α = 0.05, 1 - β = 0.80, Correlation among repeated measures = 0.5, Nonsphericity correction ε = 1, Number of groups = 3, Number of measurements = 8). Consequently, 42 participants were required for the total sample. To account for dropouts and exclusions, we planned to test a maximum of 50 individuals (16–17 in each group). The online experiment (TSST-OL) was conducted from December 18, 2023, to February 20, 2024, including confirmation of each participant’s internet environment and collection of saliva samples. Data collection for 48 participants was completed.

### Data processing and statistical analysis

The total resilience scale scores and other survey results were analyzed using one-way ANOVA for the three groups. Subjective stress and salivary hormone concentrations were analyzed using a 3 (group) × 8 (time point) repeated measures ANOVA. Individual comparisons were evaluated using a simple main-effect test and a post-hoc Holm test. The change in cortisol levels (peak value minus baseline value) from baseline (T2) to peak (T5) was calculated, and participants with a baseline-to-peak increase of less than 0.054 µg/dL were defined as non-responders [29, 30]. As a modification criterion for exploratory analysis, individuals with an increase of 15.5% or more from T2 to T5 were defined as responders [29, 30]. The chi-square test was performed to determine the prevalence of cortisol non-responders. The presence or absence of a significant difference was determined using the criterion of α = 0.05. Data from individuals whose hormonal assays showed missing values (outside the detection limits) at the eight time points were excluded from the analysis. The ω coefficients were calculated for each software using JASP version 0.16.3 [31].

### Deviation and changes from pre-registration

Pre-registration for this study was conducted before data collection in the Open Science Framework (10.17605/OSF.IO/K7EHQ; date of registration: November 17, 2023).

Individuals who had participated in previous TSST-OL experiments conducted by the author were excluded [20, 21]. In addition, exclusion criteria based on the degree of smoking habit were set a priori; however, the number of actual respondents who were smokers was low (94 out of 551); thus, adjustments were made to ensure that there were no smokers among the experimental participants.

After the main survey, participants were assigned to each group so that ages were evenly distributed; however, the overall age of respondents was unbalanced and could not be balanced (details are shown in the results section). During the experimental procedures, video-sharing time was reduced by a maximum of 3 min among participants who spent more time on saliva collection for time management. Nevertheless, the DHEA assay could not be performed in samples where saliva collection was time-consuming, and a sufficient volume could not be collected within the expected time because the cortisol assay was prioritized.

As the statistical analysis methods described in the pre-registration section were insufficient for the application of ANOVA, the data analysis methods used in this study are described below. We conducted Mendoza’s test for multi-sample sphericity, which can be applied to a mixed-design ANOVA with repeated measures [32]. When the multi-sample sphericity assumption failed, the present study used a correction by Greenhouse–Geisser’s epsilon in the case of balanced data (for subjective stress and cortisol concentrations) and a correction by Algina–Lecoutre’s Corrected Improved General Approximation (CIGA) test was used in the case of unbalanced data (for DHEA concentrations) to suppress the increase in the Type I error rate [33, 34]. We used free statistical software R version 4.0.3 [35] and R function “anovakun” version 4.8.7 [36] for ANOVA. Data on longitudinal cortisol changes as acute stress responses did not follow a normal distribution. Thus, cortisol concentrations were used in the analysis after Box–Cox transformations (X’ = (X^λ^-1) / λ and λ = 0.26) [37].

## Results

A total of 551 responses were collected in the main survey. The age distributions after the main survey were shown in Supplementary Table 2. We recruited the main survey respondents as evenly as possible in terms of age among the three groups, and 50 men were recruited for our online experiment (TSST–OL). Four dropped out because of poor internet connection or prior cancellations, and three were compensated. Of these 49 participants, one who was diagnosed with a medical illness between the survey and the experiment was excluded. Thus, data from 48 men (aged 23–69 years, mean age = 46.10 years, *SD* = 12.58) were available for data collection and analysis. To analyze salivary cortisol concentrations and subjective stress, the participants were assigned to one of three groups: No Coffee (*N* = 16, aged 23–65 years, mean age = 40.19 years, *SD* = 13.10), Low Coffee (*N* = 16, aged 24–69 years, mean age = 47.94 years, *SD* = 13.75), or High Coffee (*N* = 16, aged 33–62 years, mean age = 50.19 years, *SD* = 8.72). The mean age in each group were compared among the three groups using one-way ANOVA. No significant differences were found among groups (*F* (2, 45) = 3.02, *p* = .06, *η*^*2*^ = 0.12). The age distribution for each group was summarized in Supplementary Table 2. Demographic data of the participants are summarized in Supplementary Table 3. Shikohin consumption and sleep and exercise habits are shown in Supplementary Table 4. No participants met the exclusion criteria for missing values for the cortisol analysis. Five individuals had missing values for DHEA concentrations; the DHEA results are included as supplemental data (Supplementary Fig. 1). For other exploratory analyses, we examined the differences in sleep and physical exercise habits among the three groups.

For the time interval described on the horizontal axis of all figures, we considered the time participants took to see the video, as well as the time it took to collect saliva and to move between Zoom™ rooms. The horizontal axis is based on the average of all participants. The mean values for each interval were 30 min (T1–T2), 13 min (T2–T3), 20 min (T3–T4), 18 min (T4–T5), 13 min (T5–T6), 13 min (T6–T7), and 23 min (T7–T8).

The total resilience scale scores in each group were calculated and compared among the three groups using one-way ANOVA. No significant differences were found among groups (*F* (2, 45) = 0.47, *p* = .63, *η*^*2*^ = 0.02).

### Salivary cortisol concentrations

Among the 48 participants analyzed, 26 were responders (54%) and 22 were non-responders (46%). The number of non-responders in each group (percentage within each group) was 7 (44%) in the No Coffee group, 6 (38%) in the Low Coffee group, and 10 (63%) in the High Coffee group. A chi-square test of independence showed no difference in the prevalence of non-responders between the groups (χ^2^(2) = 2.17, *p* = .34). As a result of an exploratory analysis using the modified criteria, 35 participants were responders (73%) and 13 were non-responders (27%). The number of non-responders was 4 (25%) in the No Coffee group, 5 (32%) in the Low Coffee group, and 5 (32%) in the High Coffee group. A chi-square test showed no difference in the prevalence of non-responders between the groups (χ^2^(2) = 0.20, *p* = .90).

**Fig. 2:**
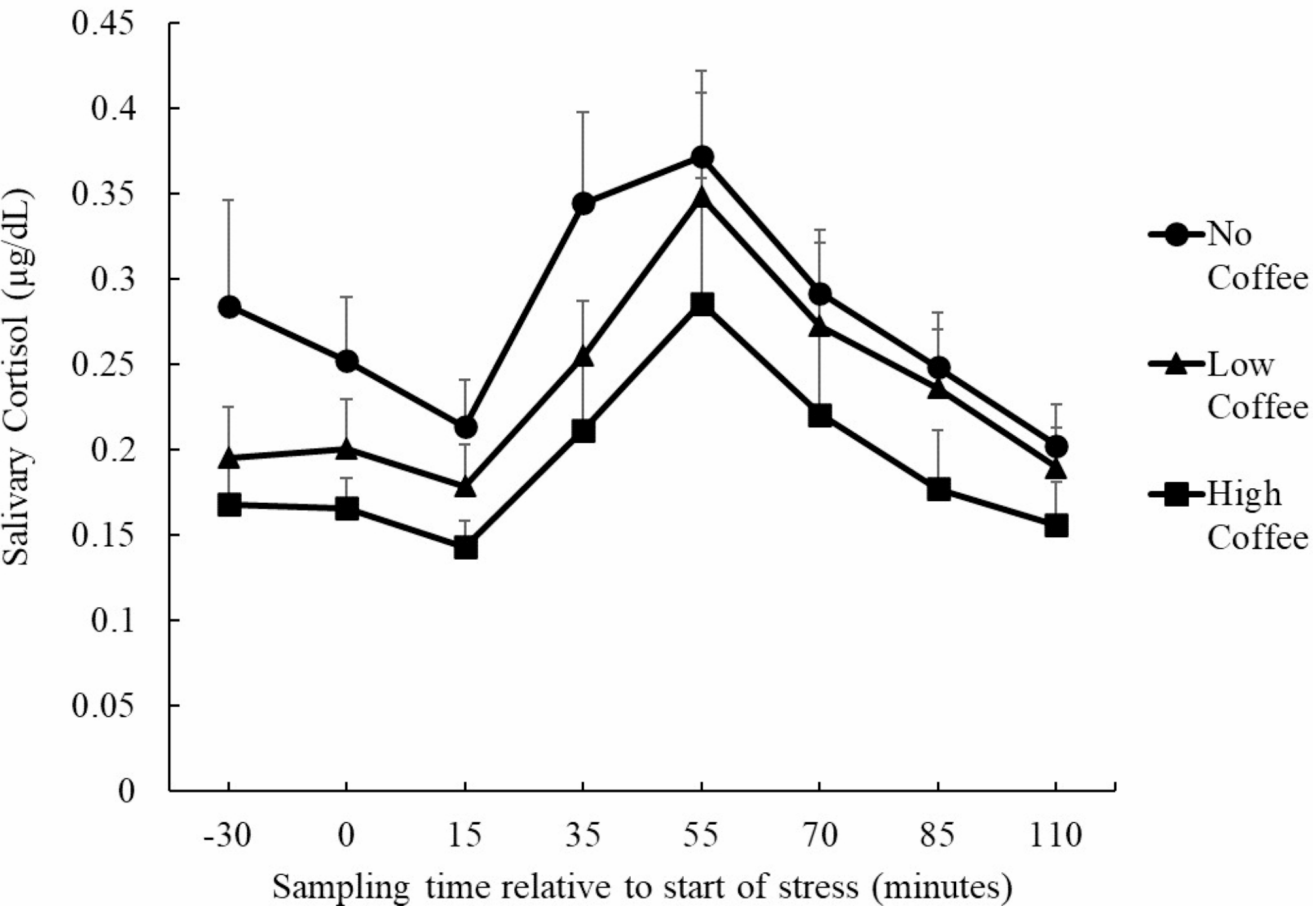
Changes in salivary cortisol concentrations during TSST-OL sessions after classification into three groups (No Coffee: *N* = 16, Low Coffee: *N* = 16, High Coffee: *N* = 16). Box–Cox transformed cortisol values were used for analysis. The graphs display the raw data values

Salivary cortisol concentrations in the three groups were analyzed using repeated-measures ANOVA (Fig. 2). As a result of Mendoza’s test, the multi-sample sphericity assumption of these data failed, and the Greenhouse–Geisser epsilon correction was performed. The main effect of time point (*F* (2.01, 90.51) = 14.61, *p* < .001, *η*_*p*_^*2*^ = 0.25) was significant. However, the main effect of group (*F* (2, 45) = 2.71, *p* = .08, *η*_*p*_^*2*^ = 0.11) and the interaction (*F* (4.02, 90.51) = 0.36, *p* = .84, *η*_*p*_^*2*^ = 0.02) were not significant. Multiple comparisons showed that the value at T5 was significantly higher than that at all other time points (*p*s < .05). In addition, the value at T2 was higher than that at T3 and lower than that at T4 (*p*s < .05). The value at T8 was lower than those at T4, T6, and T7 (*ps* < .05). The value at T3 was lower than those at T4 and T6 (*p*s < .05). The value at T7 was lower than those at T4 and T6 (*p* < .05).

### Self-rated stress

**Fig. 3:**
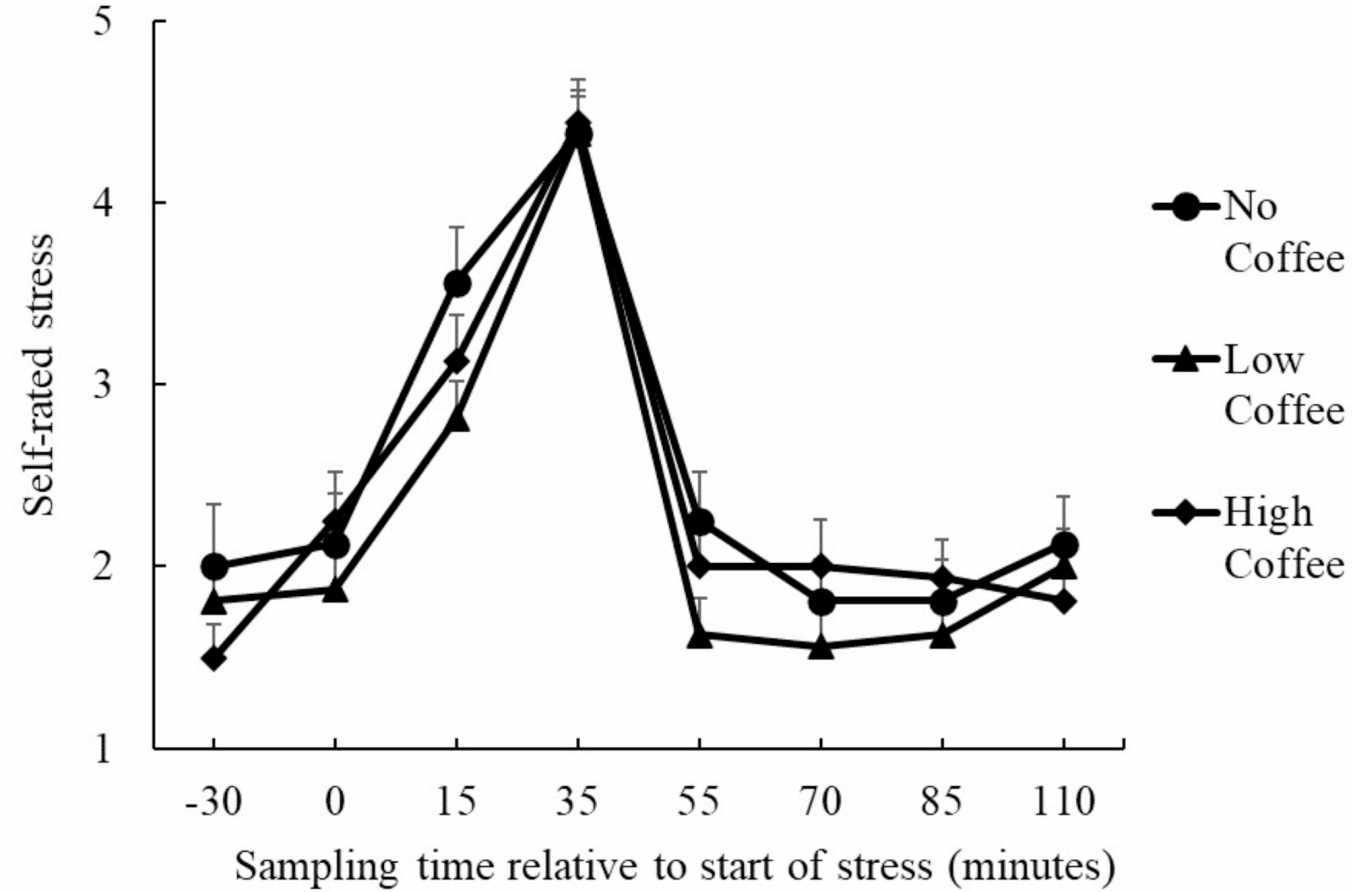
Changes in self-rated stress during the TSST–OL session after classification into three groups (No Coffee: *N* = 16, Low Coffee: *N* = 16, High Coffee: *N* = 16)

Following Mendoza’s test, the multi-sample sphericity assumption of these data failed, and the Greenhouse–Geisser epsilon correction was performed. The degree of self-rated stress in the three groups was analyzed using repeated-measures ANOVA (Fig. 3). The main effect of time point was significant (*F* (4.36, 196.26) = 78.40, *p* < .001, *η*_*p*_^*2*^ = 0.64). Multiple comparisons showed that the value at T4 was significantly higher than that at all the other points (*p*s < .05). In addition, the value at T3 was significantly higher than that at all points except T4 (*p*s < .05), and lower than that at T4. The main effects of group (*F* (2, 45) = 0.66, *p* = .52, *η*_*p*_^*2*^ = 0.03) and interaction (*F* (8.72, 196.26) = 1.11, *p* = .35, *η*_*p*_^*2*^ = 0.05) were not significant.

## Discussion

We hypothesized that the High Coffee group would show higher resilience scale scores and salivary cortisol concentrations than the other groups. The expected group differences were not clearly detected, and neither hypothesis was supported. The overall changes in salivary cortisol concentrations and subjective stress detected in the present study showed a typical pattern in TSST studies, and as in previous studies, the effectiveness and stability of the TSST-OL experiment were demonstrated [21, 27, 30].

For the TSST-OL results, the p-values and effect sizes for group differences suggested that daily coffee consumption had only a minimal influence on acute stress responses, which is consistent with the results of previous studies [7, 8]. This research plan was designed with the view that unhealthy effects could be detected in people with excessive coffee consumption, which was not the case. This could be due to loose inclusion criteria for excessive consumption. The High Coffee group in the present study may not have even been a high volume caffeine intake, based on reports that only high doses of caffeine activate the HPA axis [17]. These aspects might be more clearly investigated in populations who drink too much coffee (e.g., more than six cups a day) to be at health risk [6, 23, 38]. However, 62 out of 551 people (approximately 11%) drank more than four cups per day. Recruiting people who drink too much by setting stricter criteria for excessive consumption would be difficult because it would involve a very small population in Japan. In addition, although there was only an apparent difference, it was interesting that the High Coffee group had lower cortisol concentrations than the No Coffee group, a relationship opposite to that hypothesized. While animal model studies suggest that HPA axis responses are activated in depression-like states [39] and decreased in PTSD-like states [40], our results fit neither description. The lack of clear significant differences may be attributed to the measurement of coffee consumption habits in the present study. The lack of clear significant differences may be attributed to the measurement of coffee consumption habits in the present study. The association with habit would be more difficult to capture pronounced biological changes than with exposure to caffeine itself. Although the results were in the opposite direction of the hypotheses, they may be interpreted as indicating that coffee is less harmful to the organism.

This study excluded people who felt highly stressed daily, which may be associated with a habit of drinking high amounts of coffee. However, we should avoid mixing people in chronic stress situations with people who are not in stressful situations as participants in an acute stress experiment. Several previous studies have shown that people who are chronically stressed show altered HPA axis responses compared to those who are not. For example, previous research with burnout patients reported elevated salivary cortisol concentrations after awakening in people experiencing chronic stress [41]. Another study also reported that people at Clinical High Risk for Psychosis, characterized by chronic stress, had attenuated cortisol responses to acute stressor such as TSST [42]. Hence, people exposed to chronic stress would be affected in both their baseline and stress responses in HPA axis. In summary, we consider that the exclusion of chronically stressed individuals rather eliminated a confounding factor. Although this exclusion may have affected the sample collection of the High Coffee group, we still believe that this exclusion criterion should not be removed.

No notable differences were found in resilience scale scores. Although previous studies have reported discrepancies between questionnaires and experimental results on resilience [43–46], the present experiment provided consistent results in that coffee consumption habits were not closely related to either stress responses or resilience. Previous studies have suggested that coffee consumption reduces the risk of depression [4–6]. Taken together, these results suggest that this mechanism is not mediated by the HPA axis or personality traits.

This study has several limitations. This experiment was correlational in that it examined acute stress responses by classifying groups based on self-reports of coffee consumption habits and exposing them to psychosocial stressors, and did not examine the strong causal effects of coffee intake directly on stress responses. Additionally, the classification based only on participants’ self-reports could be biased. It may be possible to increase reliability while using self-reports by using the experience sampling method to measure coffee drinking habits in a few weeks prior to the TSST-OL and then classifying the groups based on the measurement results. In a better approach, it should be explored whether randomized assignment to groups can be used in a design such as this experiment. For example, an experiment in which non-coffee drinkers were asked to drink coffee for several weeks prior to the TSST-OL could be considered. Randomizing participants to each condition, such as the amount of coffee they drink, would reduce bias compared to this study. Futhermore, the age bias in this study cannot be dismissed, such as the absence of 20-year-olds in the High Coffee group. Since this study found that few young people who were able to participate in the TSST-OL drink a high amount of coffee, coffee-related products such as caffe lattes might be included in habit surveys, with attention to confounding [6]. As the study was designed in terms of Shikohin—a Japanese cultural concept—it did not allow for a separate discussion of the effects of coffee and caffeine. Comparisons between tea, energy drinks, and caffeine tablets may also reveal the specificity of coffee consumption. Based on the finding that the association between coffee consumption and depression and anxiety was inconsistent when elements such as decaffeinated coffee and milk were included [6], several types of coffee (decaffeinated coffee, cafe lattes, and cafe au lait) were not included in this study. Thus, our data did not cover all coffee consumption habits in Japan. Our negative results would be worth sharing, given the publication bias that positive results tend to be published [47, 48]. Moreover, the relationship between coffee and health could sometimes be exaggerated by the media and others. Our null results were obtained consistently with previous studies [7, 8] and would provide a realistic and calm perspective.

## Conclusion

The present study examined whether differences in resilience scale scores and cortisol responses to the TSST-OL could be detected depending on the extent of coffee consumption among Japanese men. Statistical analyses showed that although an effect of the TSST-OL could be found, group differences were not clearly detectable, and the hypotheses were not supported. Our results suggest that coffee consumption habits are not closely related to stress responses and resilience and might not need special weighting as a habit related to mental health.

## Electronic supplementary material

Below is the link to the electronic supplementary material.

Supplementary Material 1

Supplementary Material 2

## Data Availability

The open data on the psychological questionnaires, self-rated stress, and hormonal assays described in this article are available in the Open Science Framework (10.17605/OSF.IO/G6BXA).

## Abbreviations

ANOVA: Analysis of variance
DHEA: Dehydroepiandrosterone
HPA: Hypothalamic-pituitary-adrenal
TSST-OL: Trier Social Stress Test-Online
