# Supplementary material for: No significant difference in salivary cortisol response on the Trier Social Stress Test-Online based on coffee consumption habits
Source: BMC Psychol. 2024 Sep 13;12:483. doi: 10.1186/s40359-024-01968-3 (PMC11396474; doi:10.1186/s40359-024-01968-3)

**Supplementary Results**

**Salivary DHEA levels**

Five participants with salivary DHEA concentrations below the detection limit as a result of the EIA assays were excluded owing to missing values. The number of participants in each group was as follows: No Coffee (N = 14), Low Coffee (N = 14), and High Coffee (N = 15).

Salivary DHEA levels in the three groups were analyzed using repeated-measures ANOVA (Supplementary Figure 1). Following Mendoza’s test, the multisample sphericity assumption of these data failed, and the CIGA test was used. The main effect of time point (*F* (4.32, 123.92) = 3.92, *p* = .004, *η_p_^2^* = .09) was significant. The main effects of group (*F* (2, 40) = 0.79, *p* = .46, *η_p_^2^* = .04) and interaction were not significant (*F* (6.50, 123.92) = 0.87, *p* = .53, *η_p_^2^* = .04). Multiple comparisons showed that the value at Time Point 8 was significantly lower than that at Time Point 4 and 5 (*p*s < .05).

**Sleep and exercise habits**

Sleep hours per day and exercise frequency (days of exercise or sport per week) in the three groups were analyzed by one-way ANOVA. For sleep hours per day, the main effect was significant (*F* (2, 45) = 6.93, *p* = .002, *η^2^* = .24). Multiple comparisons showed that sleep hours in the High Coffee group was significantly shorter than those in the Low Coffee group (*p* < .05). For exercise frequency, the main effect was not significant (*F* (2, 45) = 0.21, *p* = .81, *η^2^* = .01).

Supplementary Figure 1

Changes in salivary DHEA levels during TSST–OL sessions after classification into three groups (No Coffee: N = 14, Low Coffee: N = 14, High Coffee: N = 15).


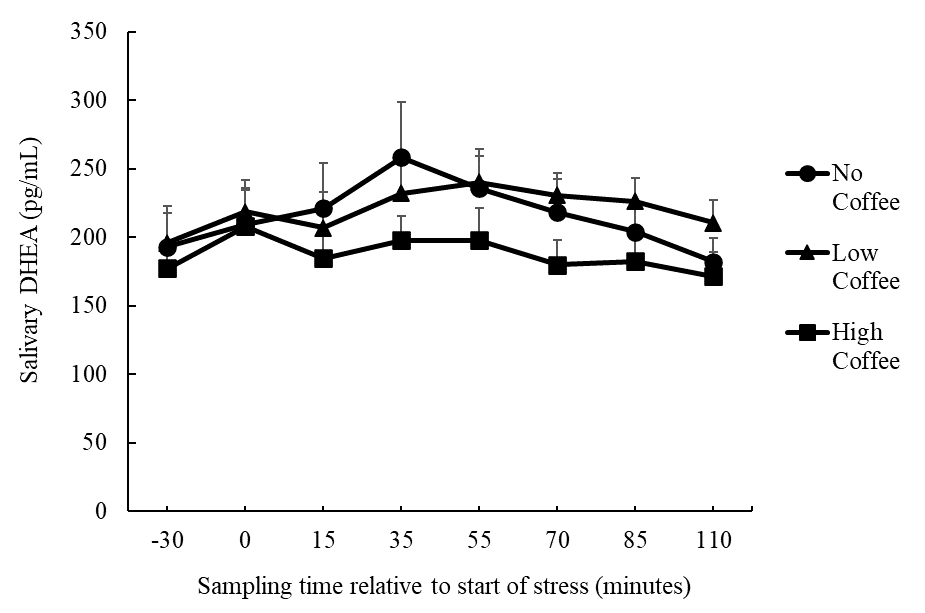

Supplement: Supplementary file 1 — Supplementary Material 1 [file 40359_2024_1968_MOESM1_ESM.docx]
